# Predicting the mechanical performance of industrial waste incorporated sustainable concrete using hybrid machine learning modeling and parametric analyses

**DOI:** 10.1038/s41598-025-11601-x

**Published:** 2025-07-20

**Authors:** Md. Alhaz Uddin, Md. Habibur Rahman Sobuz, Md. Kawsarul Islam Kabbo, Md. Kanan Chowdhury Tilak, Ratan Lal, Md. Selim Reza, Fahad Alsharari, Mohamed AbdelMongy, Masuk Abdullah

**Affiliations:** 1 Department of Civil Engineering, College of Engineering, Jouf University, Sakaka, 72388 Saudi Arabia; 2 Department of Building Engineering and Construction Management, Khulna University of Engineering & Technology, Khulna, 9203 Bangladesh; 3 HJ Russell & Company, 171 17th St NW #1600, Atlanta, GA 30363 USA; 4 Department of Software Engineering, Faculty of Science and Information Technology, Daffodil Smart City (DSC) Birulia, Daffodil International University, Savar, Dhaka, 1216 Bangladesh; 5 Department of Vehicles Engineering, University of Debrecen, Ótemető strt. 2-4, Debrecen, 4028 Hungary

**Keywords:** Sustainable concrete, Hybrid machine learning, Compressive strength, Industrial waste, Parametric analysis., Engineering, Civil engineering, Mechanical engineering

## Abstract

The construction sector is proactively working to minimize the environmental impact of cement manufacturing by adopting alternative cementitious substances and cutting carbon emissions tied to concrete. This study investigates the viability of using waste industrial materials as a replacement of cement in concrete mixes. The primary goal is to predict the compressive strength of waste-incorporated concrete by evaluating the effects of materials such as cement, fly ash (FA), silica fume (SF), ground granulated blast furnace slag (GGBFS), metakaolin (MK), water usage, aggregate levels, and superplasticizer dosages. A total of 441 data entries were sourced from various publications. Multiple machine learning techniques, such as light gradient boosting (LGB), extreme gradient boosting (XGB), and decision trees (DT), along with hybrid approaches like XGB-LGB and XGB-DT, were utilized to study how these variables influence compressive strength. The dataset was partitioned into training and testing, and statistical tools were employed to assess the correlation between input variables and strength. Model accuracy was gauged using metrics such as mean absolute percentage error (MAPE), root mean square error (RMSE), and the coefficient of determination (R^2^). Among the models, the XGB and DT approach delivered the highest precision, with an R^2^ of 0.928 in the training stage. Among hybrid models, XGB-DT exhibited a balanced performance having R^2^ value of 0.907 and 0.785 for training and testing phase. Additionally, SHAP (SHapley Additive exPlanations) and partial dependence plots (PDP) were employed to pinpoint the optimal ranges for each variable’s contribution to the improvement of compressive strength. SHAP and PDP analyses identified coarse aggregate, superplasticizers, water and cement content have high influence on model’s output. Additionally, 150–200 kg/m^3^ of GGBFS as key factors for optimizing compressive strength. The study concludes that the hybrid models along with the single models, can effectively forecast the compressive strength of concrete incorporating industrial byproducts, assisting the construction industry in efficiently evaluating material properties and understanding the influence of various input factors.

## Introduction

Concrete stands as one of the most extensively used construction materials in the construction sector. Accelerated economic advancement and urban growth have caused a notable surge in new construction endeavors, along with the deconstruction of older infrastructure^1,2^. Cement serves as a fundamental component in the creation of concrete. Nevertheless, the manufacturing industries of binders, more specifically cement, emit vast quantities of carbon dioxide, contributing to environmental degradation^3–6^. The cement sector accounts for approximately 8% of global yearly CO_2_ emissions linked to fossil fuel consumption^7–9^. Consequently, decreasing cement usage in concrete production is an urgent priority for the worldwide construction field in this century^10^. Implementing renewable and eco-friendly energy alternatives is vital for reducing carbon emissions in the cement production process. Furthermore, the depletion of natural resources and the growing deposits of industrial waste have prompted recent studies into using by-products in concrete mixtures^11^. Incorporating these discarded materials in concrete design is a contemporary approach to advancing sustainable resource practices and mitigating environmental harm, including CO_2_ emissions^12^. Combatting carbon release and reducing dependency on natural resources present major global issues that must be overcome to enable eco-production and ensure a sustainable path forward^13,14^.

In contrast, the escalating amount and complexity of industrial refuse present major difficulties for proper disposal and recycling efforts, further complicated by a lack of available disposal locations and stricter environmental mandates. Global awareness of sustainable waste handling necessitates swift action in developing and applying efficient waste treatment and disposal strategies^15,16^. Failing to manage natural resources effectively today could lead to harmful effects for future populations, stressing the urgency of improving recycling processes, finding material alternatives, and making better use of resources to minimize environmental harm^17^. The constant industrial and economic expansion worldwide has substantially boosted waste production, which correlates with the increasing demand for construction projects. This demand has led to the need for vast amounts of building materials, with concrete being the most extensively employed construction material across the globe^18^. Consequently, cement production has surged to nearly 4.1 billion tons each year, with predictions forecasting an increase to 5.8 billion tons by 2050. Addressing the ecological effects of this growth calls for both sustainable waste management practices and innovative approaches to conserving resources while ensuring responsible material production^19–23^.

Numerous methods have been developed to mitigate the detrimental effects of cement production and the environmental impacts of concrete manufacturing^24^. Increasing the incorporation of industrial waste as supplementary cementitious materials (SCMs) and other alternatives to cement clinker presents a valuable chance to lower global CO_2_ emissions and conserve natural resources^25–28^. To cut carbon emissions by one billion tonnes annually, it is necessary to substitute roughly 50% of the clinker used in cement production with alternative materials. This means integrating approximately 1.6 billion tonnes of industrial byproducts into cement production each year^29,30^. Reaching these emission reduction goals demands a multi-pronged strategy, including reducing cement content in concrete mixtures, decreasing clinker consumption in cement manufacturing, and minimizing the use of concrete in new construction projects^31,32^. Embracing these approaches will allow the cement industry to significantly lessen its environmental footprint while advancing sustainability initiatives. This includes technological innovations such as partially replacing cement with SCMs such as fly ash (FA) or ground granulated blast furnace slag (GGBFS), among others^33–35^. Additionally, the adoption of alternative additives with a smaller environmental impact is being explored to lessen the ecological damages caused by cement production^36,37^. These actions aim to enhance sustainability without compromising the performance quality of concrete^38–44^.

Evaluating the characteristics of concrete incorporating industrial byproducts typically requires extensive laboratory experimentation, which is not only expensive but also time-consuming^45^. Moreover, logistical challenges, such as limited space for curing and storing concrete specimens, often complicate thorough experimentation^46^. Concrete’s compressive strength is determined by numerous parameters, like the duration of curing, the materials incorporated in the mixture, and the type of aggregates employed, all of which necessitate detailed analysis that may prolong the testing process^47–49^. Although several empirical equations have been proposed to estimate compressive strength and improve testing efficiency, these methods are sometimes unable to determine the complex, nonlinear interactions among concrete properties and their corresponding strength^50–52^. As a result, more advanced models are required to accurately associate input variables with the performance of concrete made using industrial waste. In this context, Artificial Intelligence (AI) is demonstrated as an effective tool because of its ability to quickly learn from data, model intricate relationships, and predict results based on various input parameters^53,54^. Several studies have highlighted the success of advanced machine learning (ML) approaches in forecasting the mechanical attributes of different categories of concrete, underscoring the potential of AI for improving the predictive capability^55–57^.

To predict the fresh and hardened properties of self-compacting concrete, Support Vector Regression (SVR) with the Exponential Radial Basis Function (ERBF) kernel produced a high level of accuracy (*R* > 0.95)^58–62^. The RF model performed exceptionally well when it came to predicting the strength of recycled aggregate concrete (R^2^ = 0.97), with cement being identified as the input that had the greatest impact (32.5%)^63^. When it came to the prediction of the flexural strength of steel fiber-reinforced concrete (SFRC), GBR achieved the maximum precision (R^2^ = 0.96), outperforming both Random Forest (R^2^ = 0.94) and Extreme Gradient Boosting (R^2^ = 0.86)^64,65^. With R^2^ values of 0.92 (slump) and 0.93 (compressive strength), Multi-Expression Programming (MEP) was shown to be superior to Gene Expression Programming (GEP) when it came to alkali-activated concrete (AAC). The results of this research demonstrate the potential of machine learning models to perform accurate, efficient, and cost-effective analysis of concrete properties. Additionally, Golafshani et al.^66^ used the XGBoost approach in conjunction with the multi-objective grey wolf optimizer to estimate the elastic modulus of geopolymer concrete, and achieve high accuracy in predicting concrete parameters. This would reduce the need for experimental testing and help with the optimization of materials^67^. In addition, gene expression programming (GEP) was applied alongside artificial neural networks (ANN) to evaluate the compressive strength of geopolymer concrete containing GGBFS^68^. In a separate study, Gupta et al.^69^ implemented a multilayer perceptron (MLP) and ANN to assess the compressive strength of polymer-based composites containing FA and GGBFS. The MLP model delivered a highly accurate prediction, with an R^2^ of 0.968, effectively capturing the impact of key factors like the silica modulus, water-to-binder ratios, and curing conditions^70,71^. Khan and Abbas^72^ constructed a multilayer stacked ML model to assess the compressive strength of reactive powder concrete (RPC), establishing strong links between compressive strength and variables such as SF content, fiber proportions, and concrete age. The stacking algorithm proved the most accurate, securing an R^2^ of 0.96, followed by XGBoost at 0.954, Random Forest and ETR at 0.95, and KNN at 0.77. Similarly, Alabdullah et al.^73^ contrasted LightGBM and XGBoost models for forecasting Rapid Chloride Penetration Test (RCPT) outcomes. Their findings indicated that LightGBM outperformed XGBoost with an R^2^ of 0.9738, and SHAP analysis highlighted that compressive strength, aging, and water-binder ratio were key contributors to improving chloride resistance, with optimum performance occurring at a w/b ratio of 0.30–0.35 and 15% metakaolin replacement. Additionally, Jagadesh et al.^74^ examined the 28-day compressive strength of self-compacting concrete (SCC) using recycled aggregates, utilizing a dataset of 515 mix designs with various machine learning methods. Their evaluation revealed that the Category Boosting (CB), K Nearest Neighbors (KNN), and Extra Trees (ERT) models produced the highest R^2^ values and the lowest mean squared error (MSE), underscoring their effectiveness in accurately mapping input-output relationships^75–79^.

There are two significant gaps in existing research regarding ML techniques for forecasting the compressive strength of concrete made with industrial waste: the necessity for more advanced and accurate hybrid ML models and the scarcity of detailed parametric evaluations, such as SHAP and partial dependence plot (PDP) analyses^80^. Moreover, previous studies have generally relied on relatively small, limited datasets. Farooq et al.^81^ found that ensemble methods like boosting and AdaBoost were more effective than individual models. Shen et al.^82^ highlighted XGB as highly precise in forecasting the compressive strength of ultra-high-strength concrete. Alhakeem et al.^83^ demonstrated that gradient-boosting regression trees (GBRT) were highly reliable for evaluating the compressive strength of sustainable concrete. Uddin et al.^84^ observed that LightGBM offered greater accuracy compared to ML models such as SVR, DT and RF algorithms for evaluating the compressive strength of 3D-printed concrete. Amin et al.^85^ showed that XGBoost regressor have higher precision than SVM in assessing the compressive strength of nano-materials and fiber incorporated concrete. Khan et al.^86^ found that both RF and XGB achieved R^2^ values exceeding 0.90 when predicting the compressive strength of steel fiber-reinforced composites. Song et al.^87^ concluded that boosting methods were more accurate than neural networks and DT in estimating the compressive strength of concrete containing FA. Rahman et al.^88^ applied eleven ML algorithms to forecast the shear capacity in concrete beams, with XGB delivering the best results, evidenced by an RMSE of 1.346 and an MAE of 0.704.

This research aimed to address gaps in the current literature by applying both traditional and hybrid ML techniques, including XGB, LGB, DT, XGB-LGB, and XGB-DT models, to assess the compressive strength of concrete incorporating industrial waste. A dataset of 430 data points from prior research was compiled alongside nine key input variables, which notably improved the accuracy of the compressive strength predictions. The models’ performance was cross-validated by comparing anticipated results with actual experimental values, utilizing several statistical metrics during the training, testing, and validation stages. Furthermore, SHAP and PDP analyses were performed to explore the effect of various factors on compressive strength prediction. These analytical techniques offered valuable insights, aiding in the creation of optimized concrete mix designs with fewer trials, ultimately minimizing the need for extensive testing and reducing time, material use, labor, and associated costs.

## Methodology

### Development of ML models

#### Extreme gradient boosting (XGB) technique

XGB is an improved variation of the gradient boosting method originally developed by Friedman^89^. , utilizing multiple additive functions to improve predictive accuracy and efficiency^90^. This advanced tree-boosting technique refines gradient boosting to better handle complex datasets using-

1 $$\:\stackrel{-}{{p}_{i}}=\:{p}_{i}^{0}+\:\eta\:\sum\:_{r=1}^{N}{f}_{r}\left({Z}_{i}\right)$$

In the XGB model, the predicted outcome ($\:\stackrel{-}{{p}_{i}})\:$for the i-th data point depends on its corresponding feature set $\:{Z}_{i}$. The model utilizes N estimators, with each estimator $\:{f}_{r}$ (where r ranges from 1 to N) representing a separate decision tree structure. Initially, $\:{p}_{i}^{0}$ is set to the average of the observed values in the training data, acting as the baseline prediction. The learning rate g, also known as the shrinkage factor, progressively adjusts the model as new trees are introduced, helping to minimize the chances of overfitting, a challenge faced by most machine learning models.

The training process is iterative; at each r-th step, a new estimator is incorporated to refine the prediction from the prior step, $\:{\stackrel{-}{p}}_{i}^{\:\:\left(r-1\right)}$, with the updated prediction $\:{\stackrel{-}{p}}_{i}^{r}$ derived from the previous result and the additional estimator $\:{f}_{r}$. Overfitting is a crucial concern, and minimizing it is key to the model’s performance as

2 $$\:{\stackrel{-}{p}}_{i}^{k}=\:{\stackrel{-}{p}}_{i}^{\left(r-1\right)}+\:\eta\:{f}_{r}$$

In the XGB model, each estimator $\:{f}_{r}$ is defined by optimizing the leaf weights in the r-th tree through minimization of the objective function defined by

3 $$\:obj=\:\gamma\:E+\:\sum\:_{h=1}^{E}\left[{G}_{h}{\omega\:}_{h}+\:\frac{1}{2}\:\left({H}_{h}+\:\lambda\:\right)\:{\omega\:}_{h}^{2}\right]$$

In the XGB model, E denotes the number of leaves in the r-th tree, $\:{\omega\:}_{j}$ with (for h = 1 to E) representing the weights of these leaves. The regularization parameters r and help maintain a simpler tree structure to prevent overfitting. The parameters _h_ and _h_ are the sums of the initial and second-order derivatives of the loss function for the data points linked to the h-th leaf, aiding in balancing model complexity and predictive accuracy.

The r-th tree is generated by dividing a single leaf into multiple leaves. This process focuses on optimizing the gain parameter, which is described as

4 $$\:gain=\:\frac{1}{2}\:\left[\frac{{G}_{L}^{2}}{{H}_{L}+\:\lambda\:}+\:\frac{{G}_{R}^{2}}{{H}_{R}+\:\lambda\:}-\:\frac{{\left({G}_{L}+\:{G}_{R}\right)}^{2}}{{H}_{L}+{H}_{R}+\:\lambda\:}\right]-\:\gamma\:$$

In the model, G_L_ and H_L_ correspond to the left leaf, while G_R_ and H_R_ relate to the right leaf after a split occurs. A split is considered valid if the gain parameter is more significant than 0. Elevating the regularization parameters λ and γ, lowers the gain parameter, which contributes to keeping the tree structure simpler by limiting the complexity of leaf splits. However, this adjustment may also lower the model’s predictive capacity to accurately fit the training dataset.

#### Light gradient boosting machines (LGB) technique

The LGB technique is a well-established approach in machine learning that utilizes gradient boosting combined with decision tree methodologies^91,92^. This method incorporates a histogram-based strategy to transform continuous variables into discrete classes, facilitating the efficient analysis of extensive datasets while ensuring robust predictive capabilities, particularly for regression applications. Notable benefits of the LGB technique encompass swift model training, impressive accuracy in forecasting, consistency, minimal memory consumption, and the ability to perform training in parallel^91,92^. The LGB approach adopts a growth strategy that prioritizes leaf nodes, where the data is segmented at the leaf level, emphasizing the most significant data points during tree formation^73,93^. The ultimate result from the LGB method is achieved by averaging the forecasts from each decision tree, with information gain defined as the expected reduction in entropy stemming from node splits based on particular attributes, which can be represented as follows:

5 $$\:IG\:\left(M,U\right)=\:{F}_{n}\:\left(M\right)-\:\sum\:W\:\in\:Values\:\left(W\right)\:\frac{\left|{M}_{w}\right|}{M}\:{F}_{N}\:\left({M}_{w}\right)$$

6 $$\:{F}_{n}\:\left(M\right)=\:\sum\:_{c=1}^{B}-pc{\text{log}}_{2}pd$$

In this context, (M) denotes the overall entropy information for set M, whereas *pc* represents the fraction of M that is categorized as class c. The variable B signifies the different classes, M_w_ indicates the subset of M where the feature is equal to w, and w refers to the specific value of the attribute W.

#### Decision tree (DT) algorithm

The first model introduced is the DT technique, which is well-regarded in the literature for its adaptability in capturing intricate nonlinear relationships and its strong interpretability^94,95^. The DT method segments data into smaller groups, creating a tree-like format. This structure comprises three primary categories of nodes: (i) the root node (RN), representing the topmost point, (ii) decision nodes (DN), where conditional evaluations take place to determine how data is divided into additional subtrees based on the outcomes, and (iii) leaf nodes (LN), which function as terminal nodes that signify the final output. To assess the uniformity of the dataset, DT algorithms calculate two types of entropy measures^96^.

7 $$\:E\:\left(D\right)=\:\sum\:_{k=1}^{c}-\:{P}_{k\:}{\text{log}}_{2}{P}_{k}$$

Where P_k_ represents the fraction of dataset D that is categorized as class k.

8 $$\:E\:\left(N,Z\right)=\:\sum\:_{c\in\:X}P\:\left(c\right)\:E\:\left(c\right)$$

In this context, N indicates the target characteristic, Z corresponds to the decision characteristic, c signifies the values associated with characteristic Z, P(c) represents the chance of c occurring, and E(c) denotes the entropy linked to c.

9 $$\:Gain\:\left(N,Z\right)=E\left(N\right)-E\left(N,Z\right)$$

where N stands for the target attribute, Z signifies the decision attribute, and, c pertains to the values associated with the tuples for that attribute E(N) represents the calculated entropy of the target attribute, while E(N, Z) refers to the entropy of the tuples of Z within the N attribute^97^.

#### The hybrid ML technique

This research introduced a combined ML framework that integrates various conventional ML techniques and synthesizes the outputs from XGB, DT, and LGB algorithms. This led to the creation of two ensemble frameworks: XGB-LGB and XGB-DT, which were developed using stacking methodologies. The decision to employ hybrid techniques, specifically XGB-LGB and XGB-DT hybrid models, was driven by their demonstrated ability to enhance predictive accuracy and robustness by combining the capability of individual algorithms. Ensemble algorithms reduce the risk of overfitting and improve generalization by leveraging the complementary learning capabilities of base models. For instance, XGBoost is effective in handling complex non-linear relationships, while Decision Trees offer interpretability and low computational cost, and LightGBM is known for its high efficiency and scalability. The integration of these models thus results in a synergistic framework capable of capturing a wider range of data patterns.

### Hyperparameter tuning

The performance of the algorithms is significantly affected by hyperparameter tuning, and the selection of optimal hyperparameters is essential for the development of effective ML algorithms. The GridSearchCV class from the scikit-learn library was utilized in this research to automate the adjustment of hyperparameters in these machine learning models. Hyperparameters play a crucial role in the development of machine learning algorithms with high accuracy. GridSearchCV is an efficient and reliable technique for fine-tuning hyperparameters, although it can be time-consuming. This study examined the hyperparameters for machine learning algorithms used in this study, as presented in Table 1.

**Table 1:** Hyperparameters for ML models used in this study.

Algorithm	Parameter	Grid search range	Optimum
XGBoost	Number of estimators	[100, 200, 400]	100
	Learning rate	[0.3, 0.1, 0.01]	0.1
	Maximum depth	[3, 4, 6]	4
	Lambda	[0.1, 1, 10]	1
	Gamma	[0.001, 0.1, 1]	1
	Sample leaf	[2, 5, 10]	5
Light GBM	Number of estimators	[100, 200, 500]	200
	Learning rate	[0.2, 0.1, 0.01]	0.1
	Maximum depth	[4, 5, 6]	4
	Number of leaves	[5, 25, 30]	30
	Boosting types	[gbdt, rf, dart]	gbdt
Decision tree	Max depth	[5, 10, 20]	10
	Min samples split	[2, 20, 30]	20
	Min samples leaf	[3, 5, 10]	5
	Maximum features	[sqrt, log2]	sqrt
	Criterion	[squared_error, friedman_mse]	squared_error
	Splitter	[best, random]	best
	Max leaf nodes	[10, 20, 50]	50

### Parametric analysis

#### Shapley additive explanations (SHAP)

SHAP interprets a model’s predictions by expressing them as a linear combination of input features, facilitating a clear insight into how each variable influences the final outcome^98–100^. This methodology is grounded in game theory, specifically the Shapley values, which assist in identifying the contribution of each participant in a collaborative setting^91^. In the realm of machine learning, the input features act as these participants, while the model’s outputs represent the overall “game”. SHAP determines the significance of each feature by observing variations in predictions when specific features are included or excluded from the input set, thereby providing a comprehensive scenario of how each feature affects the model’s results.

10 $$\:z\left(t{\prime\:}\right)=\:{\varphi\:}_{o}+\:\sum\:_{j=1}^{M}\left({\varphi\:}_{j}\:{X}_{j}^{{\prime\:}}\right)$$

Here, the Shapley values $\:{\varphi\:}_{j}$ function as the coefficients in this linear setup, where t′ represents a generalized version of the original input vector t, and z stands as the interpretative model. These coefficients provide insights into the contributions of individual features to the overall prediction.

11 $$\:{\varphi\:}_{j}=\:\sum\:_{G\subseteq\:H\backslash\:\left\{j\right\}}\frac{\left|G\right|!\:\left(\left|H\right|-\:\left|G\right|-1\right)!}{\left|H\right|!}\:\left[{f}_{SU\left\{j\right\}}\:\left({X}_{SU\left\{j\right\}}\right)-\:{f}_{s}\:\left({X}_{s}\right)\right]$$

In this case, H represents the full collection of input features, and G refers to a subset derived from H, excluding the feature associated with index j.

#### Partial dependence plots (PDP) method

Single-dimensional Partial Dependence Plots (PDPs-1D) are highly effective for depicting how one particular input variable correlates with the predicted result. In general, PDPs shed light on the significance of each input factor in influencing the model’s predictions. As a holistic tool, they analyze all instances to expose the broader link between a feature and the projected outcome^101^. These visualizations illustrate how individual input factors shape the predictions by showing the influence of varying their values on the machine-learning model^102^. Additionally, PDPs uncover the relationship between specific input features and the forecasted outcomes, enhancing the understanding of each feature’s role in shaping the final prediction.

### Evaluation metrics for prediction performance

The prediction accuracy of the algorithms was assessed using three distinct statistical methods employed at three stages: training, validation, and testing. These statistical performance determinants are root mean square error (RMSE), mean absolute error (MAE), and mean absolute percentage error (MAPE). Additionally, the coefficient of determination (R^2^) is used to evaluate how well the prediction coincides with the experimental outcome. RMSE helped to calculate the typical deviation between actual and predicted values, while MAPE assessed the percentage discrepancy between them.

12 $$\:RMSE=\:\sqrt{\frac{1}{m}}\:\sum\:_{i=1}^{m}{\left({Q}_{i}-\:{R}_{i}\right)}^{2}$$

13 $$\:MAPE=\:\frac{1}{m}\:\sum\:_{i=1}^{m}\left|\frac{{Q}_{i}-\:R}{{R}_{i}}\right|$$

14 $$\:{R}^{2}=1-\:\frac{\sum\:_{i=1}^{m}{\left({Q}_{i}-\:{R}_{i}\right)}^{2}}{\sum\:_{i=1}^{m}{\left({Q}_{i}-\:{\mu\:}_{i}\right)}^{2}}$$

**Fig. 1:**
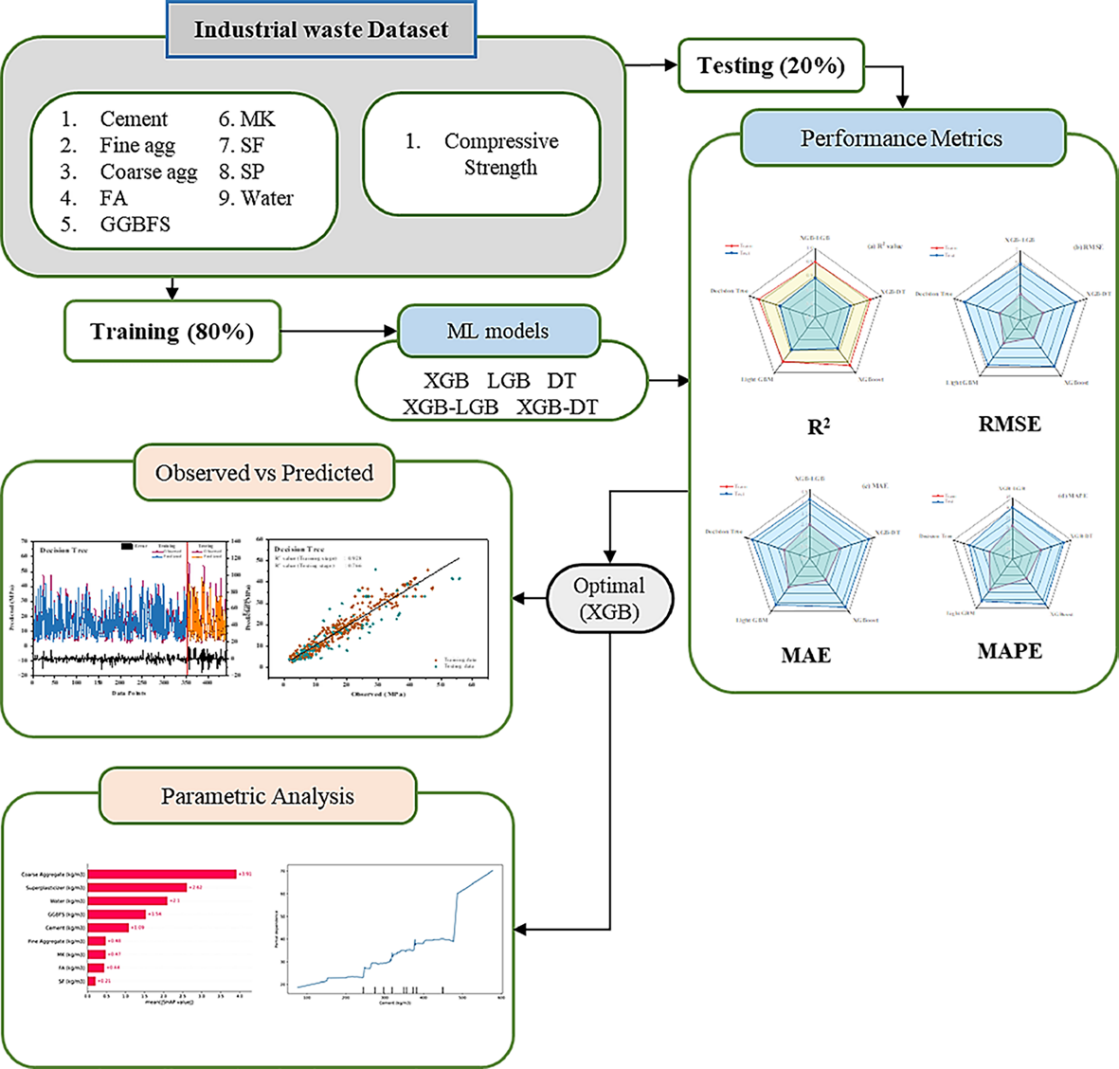
Methodological flow chart.

In this context, $\:{Q}_{i}$ indicates the observed values, $\:{R}_{i}$ signifies the predicted values, $\:{\mu\:}_{i}$ i represents the average, and m corresponds to the total data points. Figure 1 illustrates the overall methodological flowchart used in this study.

## Results and discussion

### Analysis of dataset

**Fig. 2:**
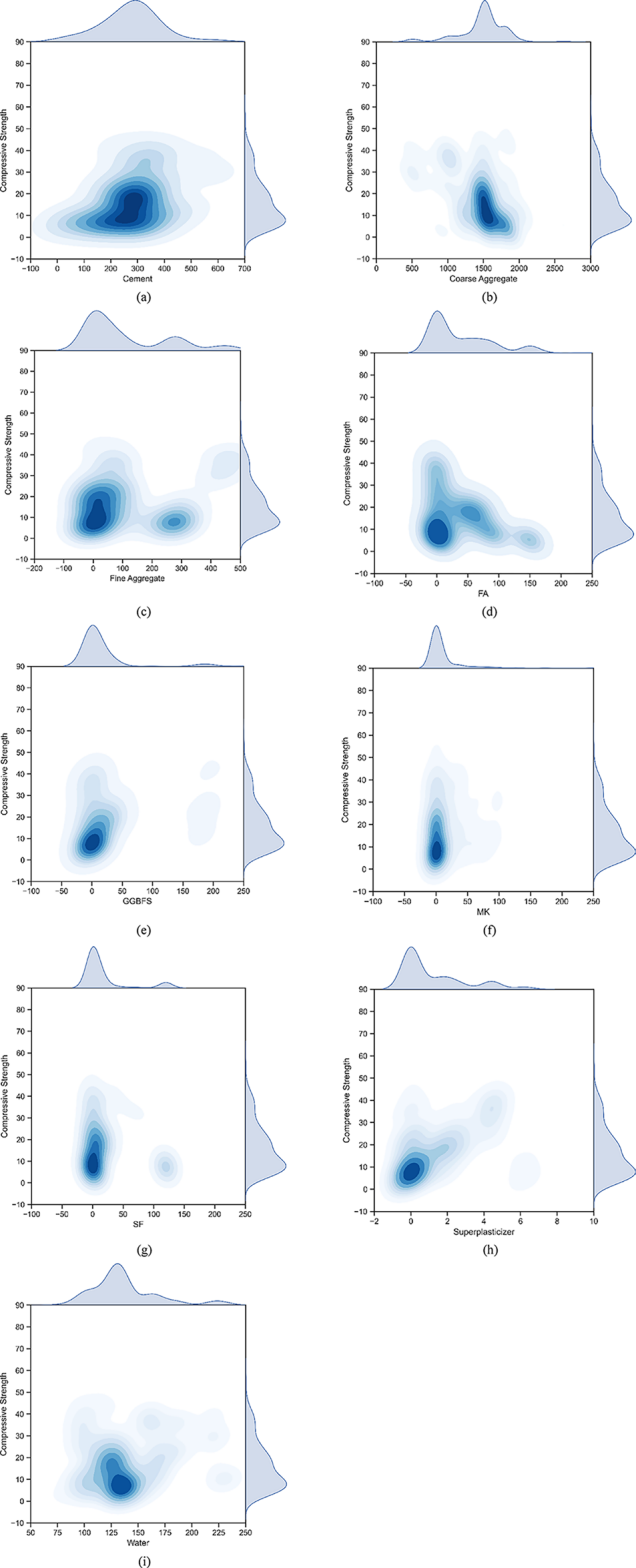
Contour map of input and output variables.

In this research, a detailed dataset consisting of 441 records was compiled from various sources in the literature. The concrete mixtures studied involved the use of multiple industrial by-products, such as FA, SF, GGBFS, and MK, in combination with ordinary Portland cement, aggregates, SP, and water. The critical input variables encompassed the quantities of cement, FA, SF, GGBFS, MK, curing time, water amount, aggregate volume, and superplasticizer concentration. Table 2; Fig. 2 combined offer a clear overview of the input and output data, as well as their statistical characteristics. The table outlines various statistical metrics for each parameter, such as minimum and maximum values, standard deviation, and coefficient of variation (COV).

**Table 2:** Descriptive analysis of the input concrete ingredients.

Input materials	Unit	Minimum	Maximum	Mean	Deviation	COV
Cement	kg/m^3^	90	1370	300.55	194.53	64.73
Coarse Aggregate	kg/m^3^	506	2649	1487.70	312.01	20.97
Fine Aggregate	kg/m^3^	0	467	110.45	141.54	128.15
FA	kg/m^3^	0	247	39.7	51.39	129.45
GGBFS	kg/m^3^	0	247	11.42	30.38	266.03
MK	kg/m^3^	0	420.1	17.74	51.87	292.39
SF	kg/m^3^	0	120	17.05	36.72	215.35
Superplasticizers	kg/m^3^	0	6.3	1.21	1.74	143.92
Water	kg/m^3^	78	230.05	137.13	30.43	22.19
Compressive strength	MPa	1.5	55.73	16.43	11.09	67.51

Figure 2 illustrates the association between compressive strength values and various concrete mix components. Figure 2(a), cement content shows a high concentration between 300 and 400 kg/m^3^. For coarse aggregate, as shown in Fig. 2(b), the data is predominantly clustered between 1200 and 2000 kg/m^3^, while fine aggregate data in Fig. 2(c) is primarily between 0 and 300 kg/m^3^. The relationship between compressive strength and industrial waste content, as shown in Fig. 2(d-g), is more complex, reflecting variability in the proportions of materials like FA, SF, and GGBFS from different studies. In Fig. 2(h), superplasticizer (SP) shows minimal variation, with around two-thirds of the data reporting quantities below 6 kg/m^3^. Lastly, Fig. 2(i) indicates that the water content associated with compressive strength is generally between 100 and 150 kg/m^3^.

### Pearson correlation matrix

**Fig. 3:**
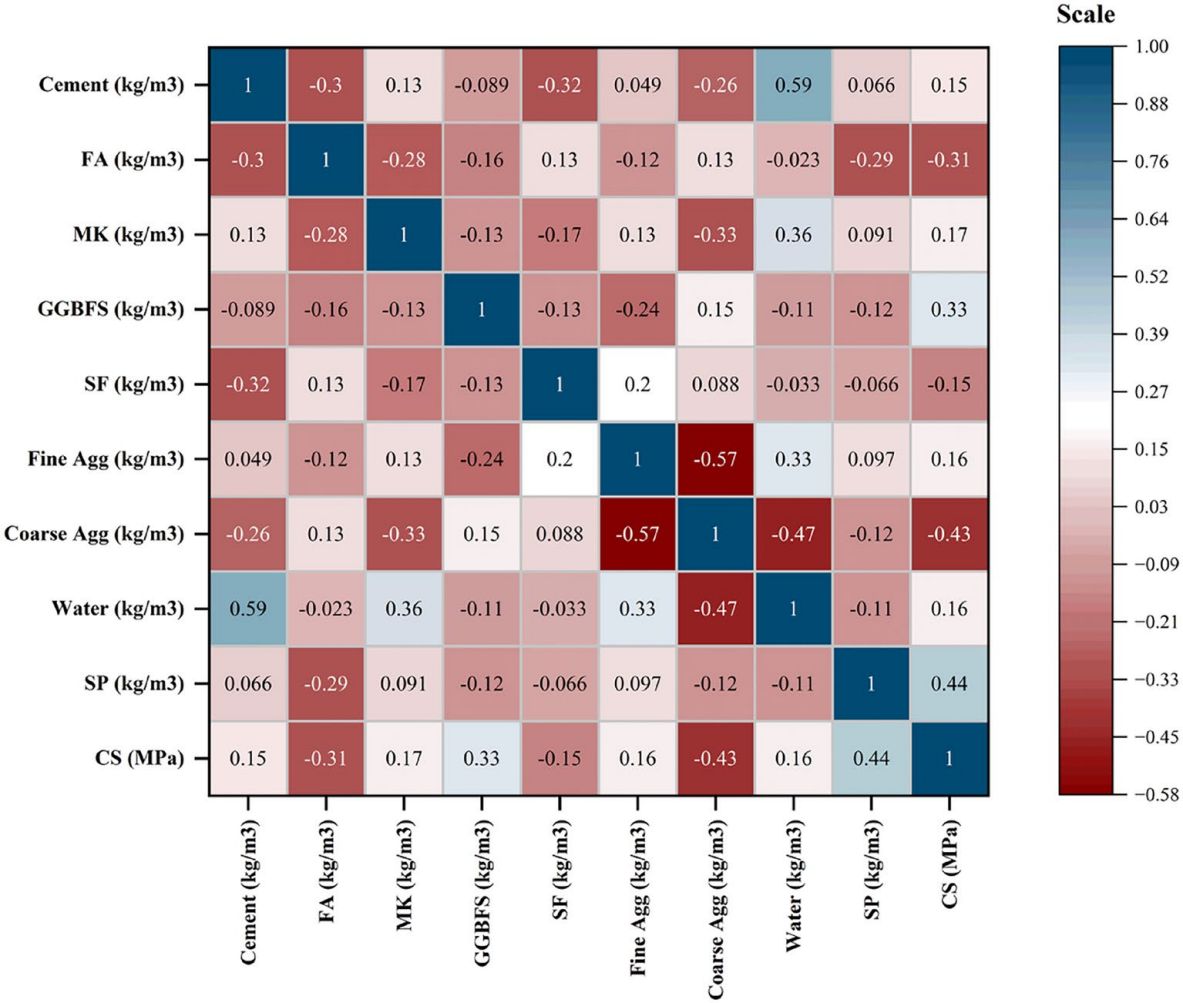
Matrix depicting the correlation between input and target parameters.

The correlation coefficient varies between − 1 and 1, with values exceeding 0 indicating a positive relationship and negative values reflecting the opposite trend. A higher absolute coefficient value suggests a stronger connection. Figure 3 illustrates the correlation coefficients between input and output variables, which range from − 0.57 to + 0.59. The heat map indicates that six input variables have a positive interaction with compressive strength, with the exception of water content and aggregate. The relationship between cement and compressive strength is relatively weak but positive (*R* = 0.15), suggesting a moderate level of consistency with strength. Metakaolin (MK), GGBFS, fine aggregate, water, and superplasticizer (SP) also show positive correlations with compressive strength, with respective R values of 0.17, 0.33, 0.16, 0.16, and 0.44. Conversely, a moderate negative correlation exists between SF and FA concerning compressive strength, indicating that strength diminishes as the contents of FA and SF rise. A similar negative association is noted for coarse aggregate.

### XGB model performance assessment

**Fig. 4:**
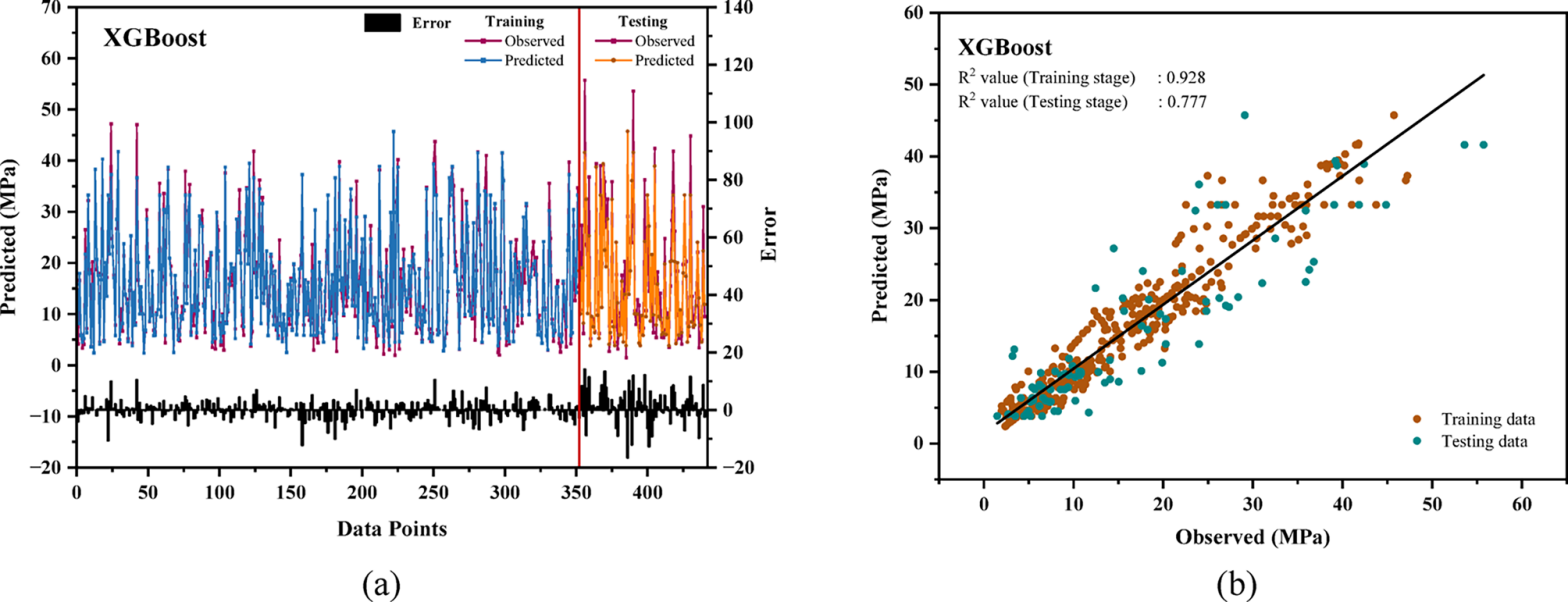
(**a**) The relative error in compressive strength predictions using the XGB model for train-test datasets. (**b**) The linear relationship between predicted and actual compressive strength of the XGB model.

Figure 4 showcases the outcomes of applying the XGB algorithm to forecast compressive strength. In Fig. 4(a), a combination of a line graph and scatter plot demonstrates the correlation between predicted and actual values for both the training and testing datasets. The prediction error for the training dataset remains below 6 MPa, while for the testing data, the error margin is approximately 20 MPa. Figure 4(b) highlights the relationship between actual and predicted values, showing R^2^ values of 0.928 for the training data and 0.777 for the testing data. This indicates a high prediction accuracy for the training dataset and moderate accuracy for the testing set. The XGB model demonstrated high accuracy in predicting the compressive strength of waste marble powder concrete (R^2^ > 0.97), indicating its reliability for optimizing WMP concentration in concrete mixtures^103^.

### Evaluation of the performance of the LGB model

**Fig. 5:**
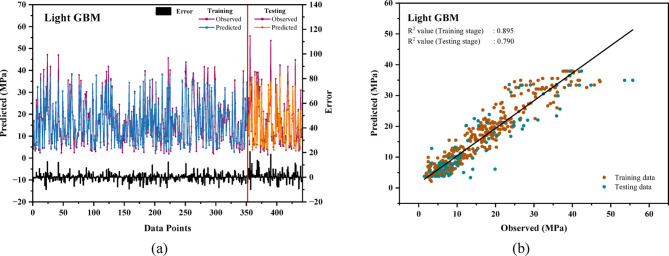
(**a**) Relative error of compressive strength in the LGB model for train-test datasets. (**b**) The linear relationship between predicted and actual compressive strength of the LGB model.

Figure 5(a) displays the findings from the training and testing stages of the LGB model. The highest error observed is around 15 MPa in the training stage and about 20 MPa in the testing stage. The error range during the training phase is broader for the LGB model when compared to the testing data of the XGB model, although the testing errors for both models are similar. In Fig. 5(b), the R^2^ values for the LGB algorithm are 0.895 for training and 0.79 for testing.The LGB model demonstrated consistent performance in predicting the CS of Recycled Aggregate Concrete, attaining an impressive Coefficient of Determination (R^2^) value of 0.88, underscores its potential as a powerful tool in sustainable construction analysis^104^. Overall, the XGB model shows a better alignment between predicted and actual data than the LGB model.

### Evaluation of the performance of the DT model

**Fig. 6:**
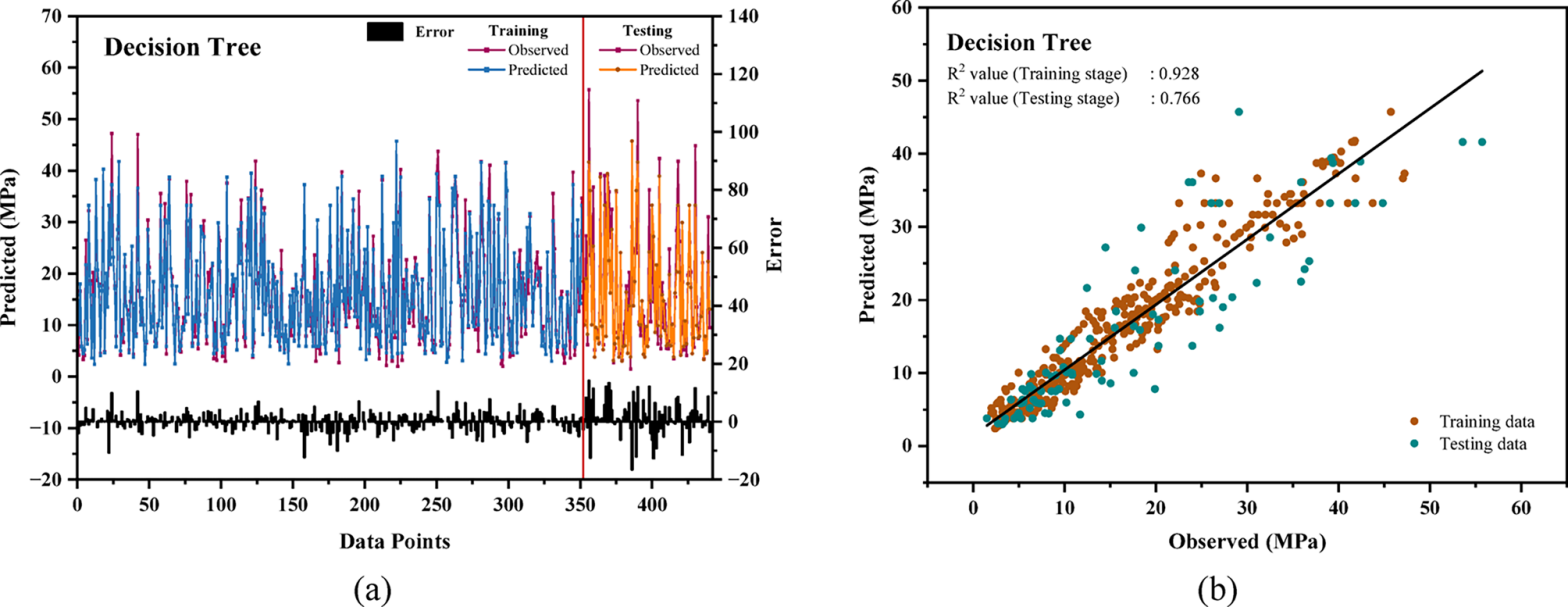
(**a**) Relative error of compressive strength for the Decision Tree (DT) model for train-test datasets. (**b**) The linear relationship between predicted and actual compressive strength of the DT model.

Figure 6(a) displays the outcomes from the training and testing phases for the Decision Tree (DT) model, indicating a maximum error of approximately 20 MPa for both datasets. The training phase for the DT model exhibits a higher error compared to the XGB and LGB models, while the testing errors remain consistent across all models. Figure 6(b) presents the R^2^ values for the DT model, which are 0.928 for the training set and 0.766 for the testing set. The DT model, which achieved an impressive average accuracy of 88.32%, demonstrated its effectiveness as a dependable and potent instrument for predicting construction waste, paving the way for more sustainable construction practices and intelligent waste management^105^. Among the individual models (XGB, LGB, and DT), the DT model demonstrates similar prediction performance on both new and existing datasets, although the XGB model achieves superior accuracy overall.

### Evaluation of the performance of the hybrid model

**Fig. 7:**
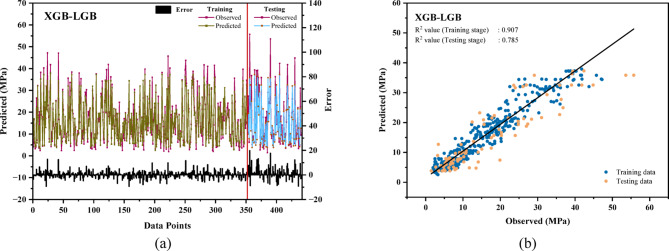
(**a**) Relative error in compressive strength for the XGB-LGB model for train-test datasets. (**b**) The linear relationship between predicted and actual compressive strength of the XGB-LGB model.

**Fig. 8:**
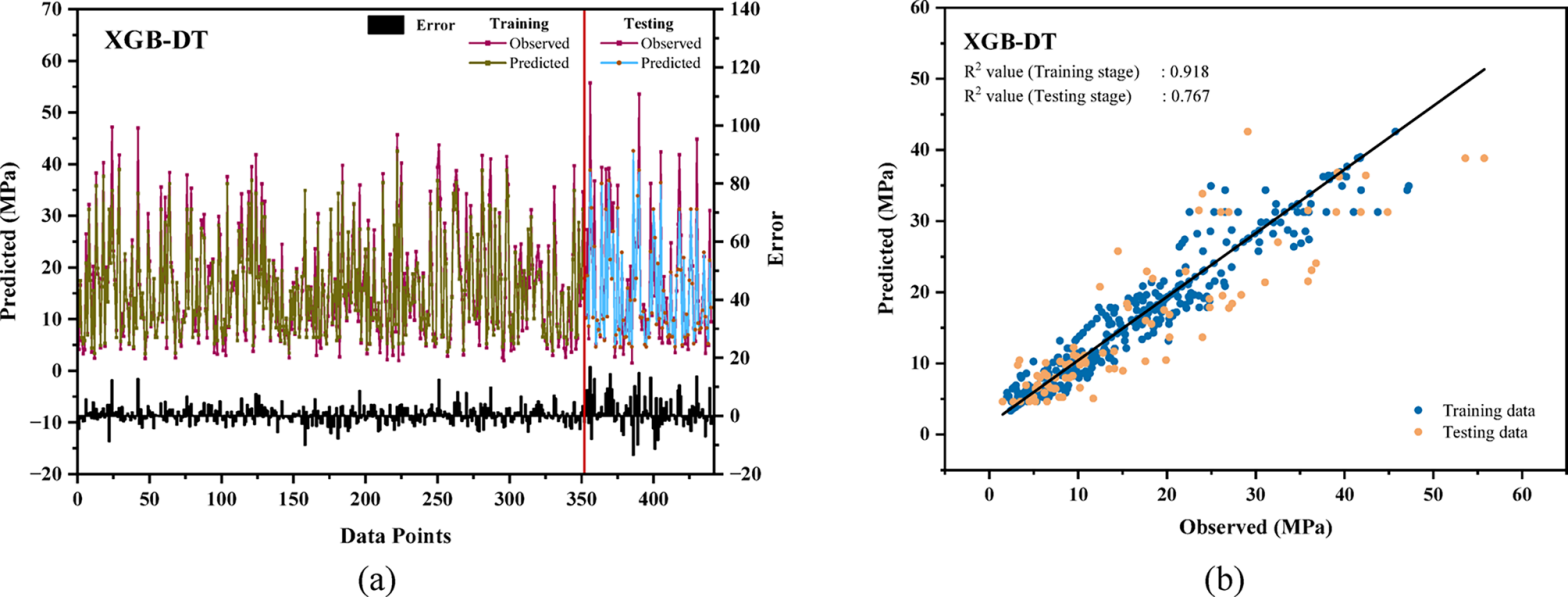
(**a**) Relative error in compressive strength for the XGB-DT model across training and testing datasets (**b**)Linear relationship between predicted and actual observations in the XGB-DT model.

The hybrid models, XGB-LGB and XGB-DT, exhibited superior performance compared to their individual versions. In Fig. 7(a), the XGB-LGB model records a reduced error, with testing errors remaining below 8 MPa and training errors under 20 MPa. Similarly, Fig. 8(a) illustrates a comparable range of errors for the XGB-DT model. The regression coefficients for both models, shown in Figs. 7(b) and 8(b), indicate greater accuracy than the standalone models, highlighting their enhanced predictive capability. The hybrid XGB-LGB model exhibited exceptional accuracy (R^2^ = 0.95, RMSE = 5.255 MPa) in predicting the CS of rice husk ash-based concrete, underscoring its potential as a precise and efficient instrument for sustainable construction applications^106^.

### Evaluation of the performance of developed models

**Fig. 9:**
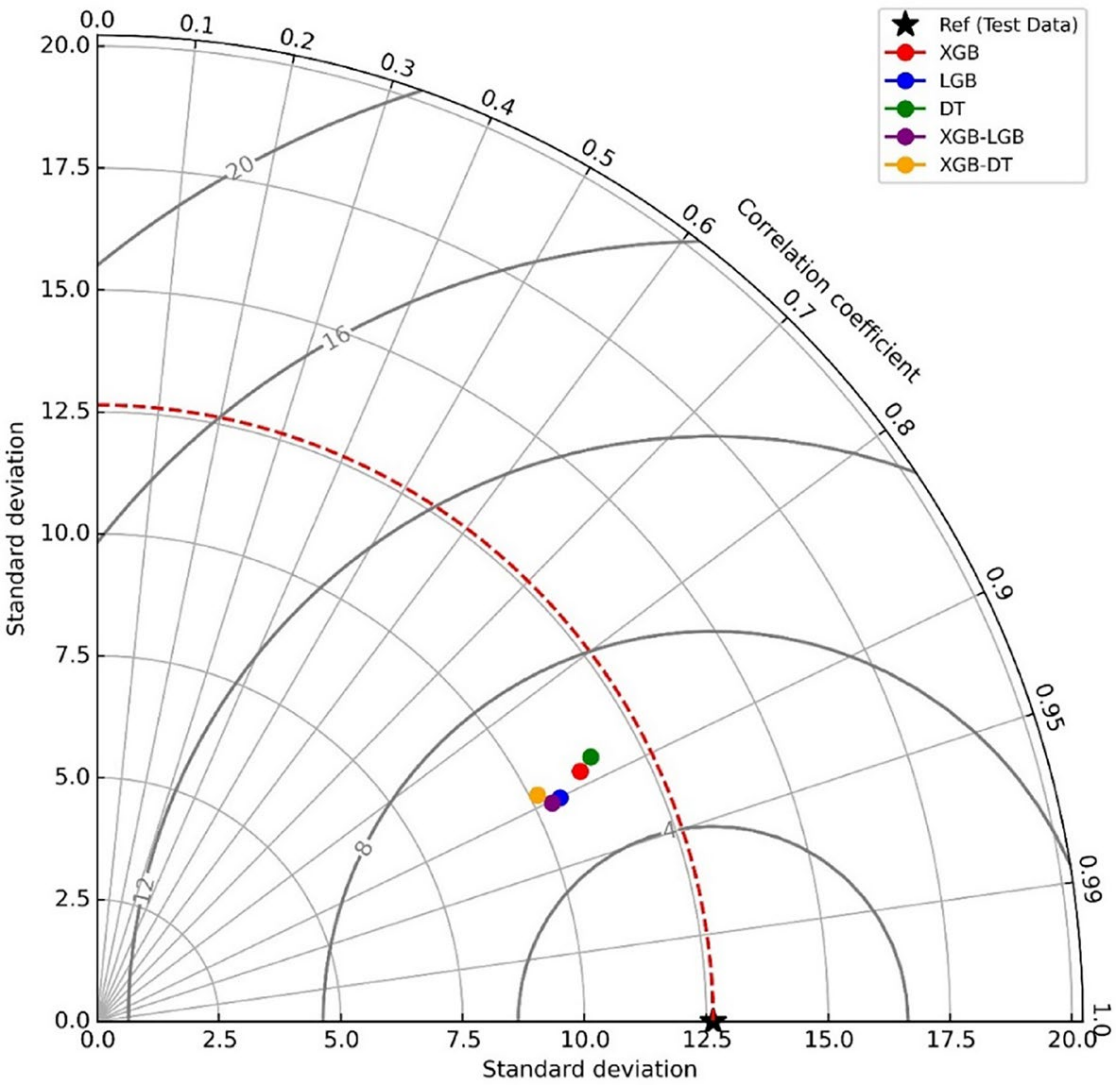
Taylor diagram for evaluating the effectiveness of ML models used in this study.

The Taylor diagram provides an effective visual aid for comparing the performance of different models with experimental data. It estimates how well the predicted values align with the experimental values by determining the correlation coefficient (R^2^) and standard deviation. An R^2^-value of 1 indicates that the anticipated values match the observed values perfectly in terms of relationship and variability. Figure 9 illustrates a Taylor diagram for five training models that demonstrate a correlation coefficient higher than 0.7, indicating relatively strong alignment with the train data, although slight differences can be noted in their variability. The standard deviation ranges from approximately 10 to 12.5, with the XGB and DT models positioned close to the reference point, suggesting they have higher precision.

The XGB-DT model, while showing a lower correlation coefficient, also exhibits a slightly elevated standard deviation compared to the XGB and DT models, reflecting its relatively lower consistency in prediction. Conversely, the XGB-LGB combination demonstrates strong performance with a slightly better correlation than some other models. Overall, the XGB, DT, and XGB-LGB models appear to balance correlation and standard deviation, indicating their close alignment with the reference data and solid predictive performance.

**Fig. 10:**
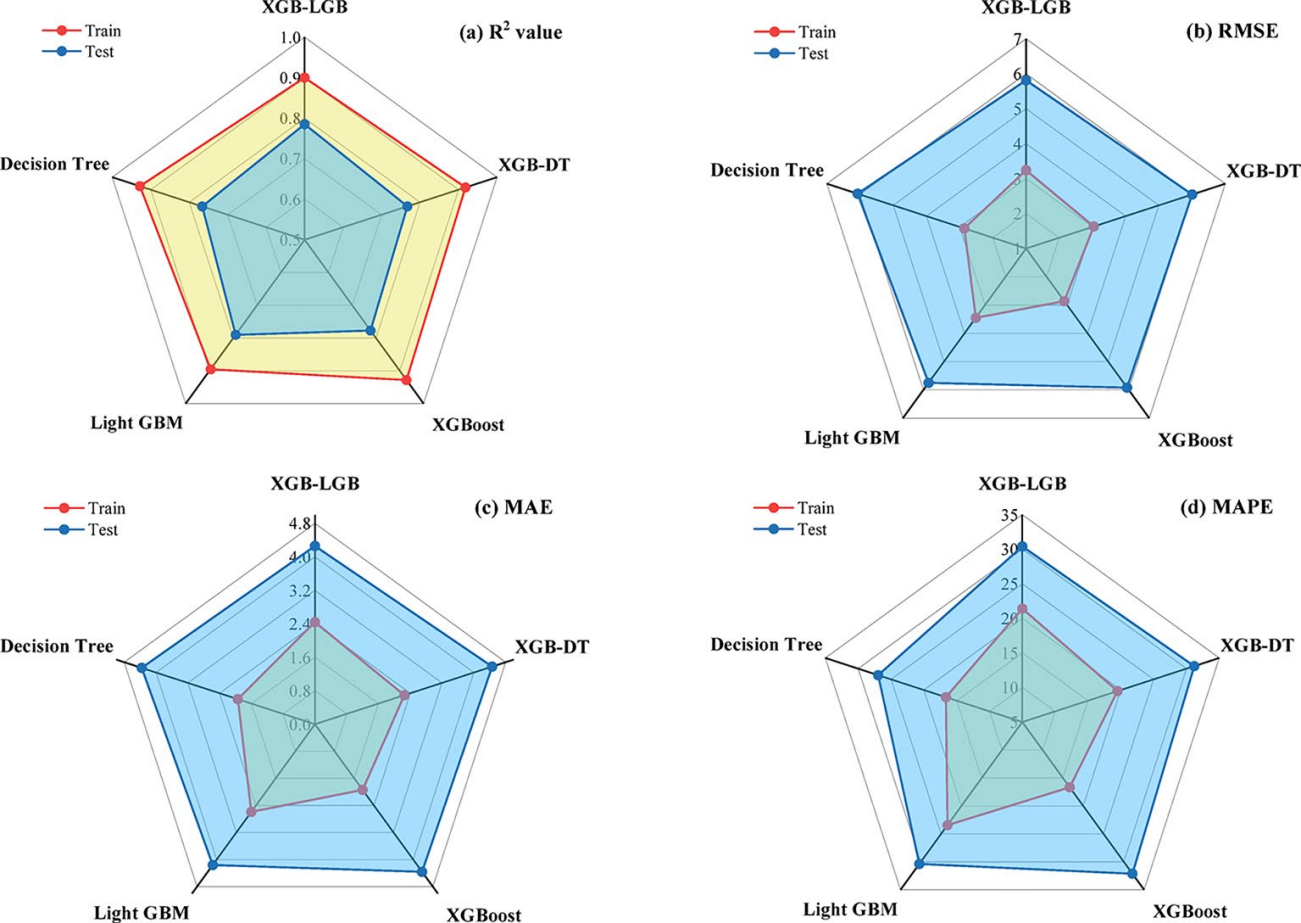
Performance of ML models.

Figure 10 displays a radar chart that evaluates various machine learning models according to R^2^, RMSE, MAE, and MAPE for both training and testing datasets. In Fig. 10(a), the XGB and DT models exhibit the strongest performance, achieving R^2^ values of 0.928 for training both models and 0.777 and 0.766 for testing, respectively. In contrast, the LGB model recorded the lowest R^2^ values, with 0.895 for training and 0.79 for testing. Among the two hybrid models, XGB-DT demonstrates the strongest performance during training with an R^2^ value of 0.917, while XGB-LGB achieves the best performance during testing with an R^2^ value of 0.785, which is closest to the highest testing value. Figure 10(b) indicates that the DT model achieved the smallest RMSE for both datasets, at 2.8634 for training and 6.0766 for testing, while the LGB model had the highest RMSE, XGB-DT also exhibits lower RMSE values compared to XGB-LGB. In Fig. 10(c), the DT model once again displayed the lowest MAE for testing at 1.9416. MAE value of 1.945 and 2.25 for XGB and XGB-DT, respectively, whereas the LGB model reported the highest at 2.5967. Lastly, Fig. 10(d) illustrates the DT model with the least MAPE at 16.6413 for testing, while the LGB model had the highest at 23.4427. Overall, the XGB and DT models surpassed the others, demonstrating superior R^2^ values and reduced error rates, whereas, among the hybrid models, XGB-DT achieved the best performance.

Furthermore, the time efficiency of each analytical approach plays a critical role in practical implementation. In this study, training durations for all models were monitored, and it was observed that the DT model offered the shortest computational time due to its simpler structure, while the XGB-LGB hybrid model required the longest training period, reflecting the computational complexity of ensemble techniques. Despite the slightly extended training time, the hybrid models (XGB-LGB and XGB-DT) demonstrated improved accuracy, justifying the trade-off between time and performance. In addition, the bossting-based algorithms (XGB and LGB) required prolonged time comparing DT for processing and predicting the CS of modified concrete.

### SHAP analysis

**Fig. 11:**
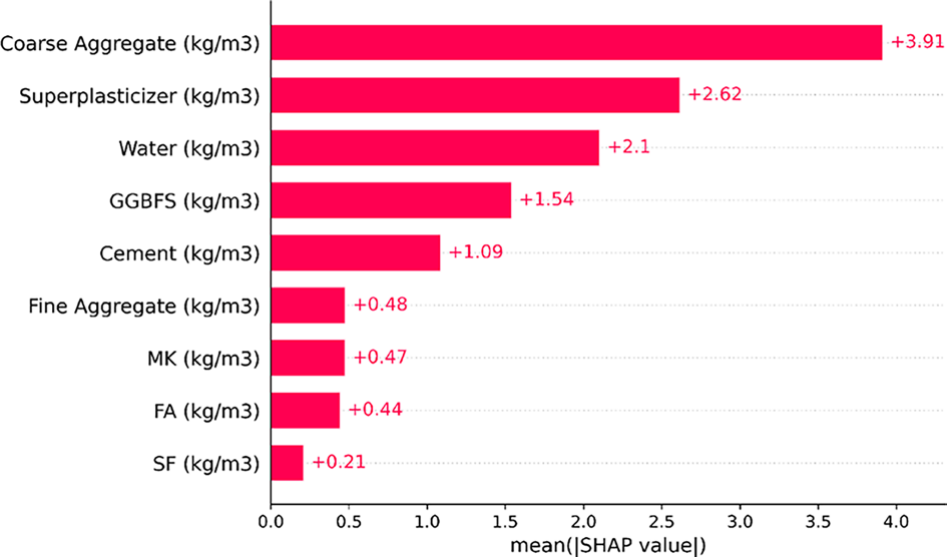
Mean values of SHAP.

Identifying the essential elements for precise model predictions is essential. This study utilized mean SHAP values to assess the importance of each input variable. Figure 11 depicts the average SHAP values for the input features of the XGB model. Coarse aggregate was found to have the most significant influence on the model’s predictions, with a SHAP value of 3.91. Superplasticizer ranked as the second most important factor influencing concrete compressive strength. Among the binders, GGBFS contributed the most, followed by MK, FA, and SF, all of which improved strength by enhancing the concrete’s microstructural stiffness. Water showed a higher SHAP value than fine aggregate, though both were among the less significant factors. The SHAP analysis demonstrated that chloride resistance is substantially influenced by compressive strength, aging, and the water-binder ratio. The optimal performance was achieved with a water-binder ratio of 0.30–0.35 and a 15% metakaolin replacement, which ensured enhanced durability against chloride penetration^73^.

### Overview diagram of SHAP effect metrics

**Fig. 12:**
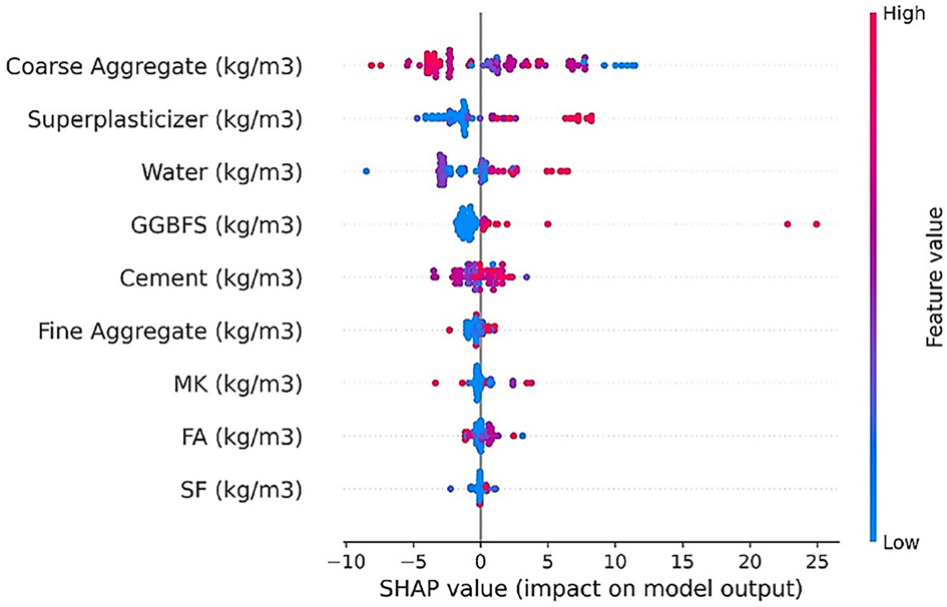
SHAP impact values.

The application of SHAP game theory facilitated the ranking of the significance and predictive influence of all input variables. SHAP quantifies the average marginal effect of each variable over all possible combinations. The relative importance of each variable was computed and ranked in descending order to emphasize the overall feature relevance. Figure 12 illustrates the significance of each variable through colored points, with the Y-axis indicating feature importance and the X-axis representing SHAP values. This graph demonstrates the relationship between each input and its corresponding SHAP value. For example, cement exhibited a notable SHAP value, signifying a positive contribution to concrete compressive strength. Conversely, coarse aggregate displayed a negative correlation between its SHAP value and feature values. SP and GGBFS exhibited moderate relevance, whereas SF and FA were ranked lower. MK and fine aggregate, despite their moderate SHAP values, had a beneficial effect on concrete strength. An expanded dataset would offer clearer insights into the patterns and effects of each input.

### SHAP heat map

**Fig. 13:**
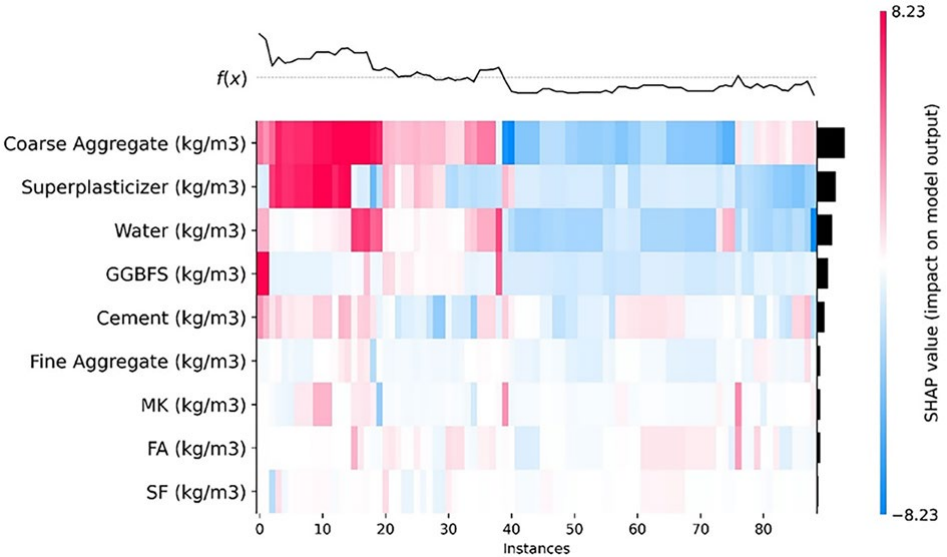
SHAP heat map.

Figure 13 displays a heat map depicting SHAP values for different features across individual data instances. The f(x) graph showcases the fluctuations in SHAP values among data points with comparable f(x) scores. In the initial 40 instances, coarse aggregate and SP exhibit significant SHAP magnitudes; however, from data points 40 to 90, the SHAP values turn negative for coarse aggregate. Coarse aggregate displays a higher SHAP value in the initial 40 cases, which declines for the remaining points. Other features consistently exhibit lower SHAP values, likely due to data limitations in past studies. This research aims to fill that gap, offering insights for developing more accurate predictive models.

### investigation of SHAP interaction graphs

**Fig. 14:**
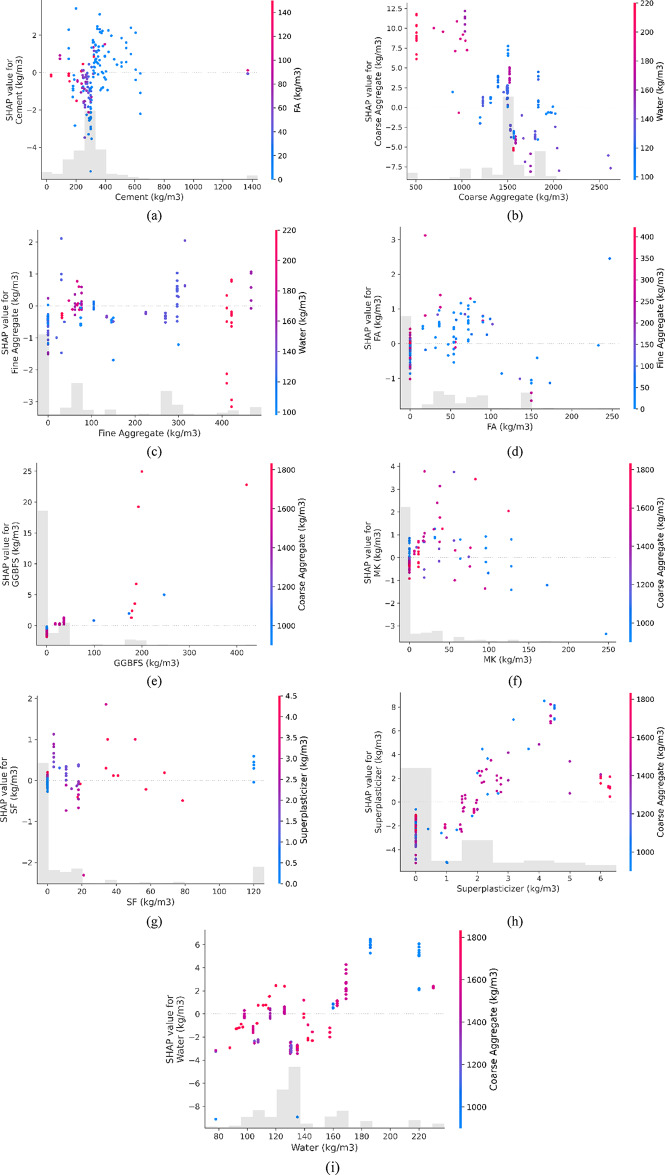
Interaction plots for all input parameters.

Figure 14 illustrates the relationships among input variables and their impact on compressive strength. In Fig. 14(a), the relationship between cement and FA strengthens as cement content increases. Figure 14(b) highlights that coarse aggregate and water have a greater impact, up to 1000 kg/m^3^, after which the influence decreases. In Fig. 14(c), the interaction between fine aggregate and water shows a moderate correlation up to 100 kg/m³, with a higher impact above 400 kg/m^3^. According to Fig. 14(d), FA negatively affects compressive strength, suggesting that keeping FA between 10 and 100 kg/m^3^ yields better strength. In Fig. 14(e), using around 200 kg/m^3^ of GGBFS with coarse aggregate positively impacts compressive strength. Figure 14(f) shows that an optimal MK content between 10 and 50 kg/m^3^ improves compressive strength with coarse aggregate. Figure 14(g) shows SF having a stronger effect at around 40 kg/m^3^ when combined with SP, and Fig. 14(h) highlights that SP performs best with coarse aggregate in the 3 to 5 kg/m^3^ range. Finally, Fig. 14(i) demonstrates that water significantly influences compressive strength as coarse aggregate increases. The outcomes of this SHAP analysis are consistent with the results reported by Li et al.^107^, which details the beneficial and detrimental influences on the strength of concrete.

### PDP analysis

**Fig. 15:**
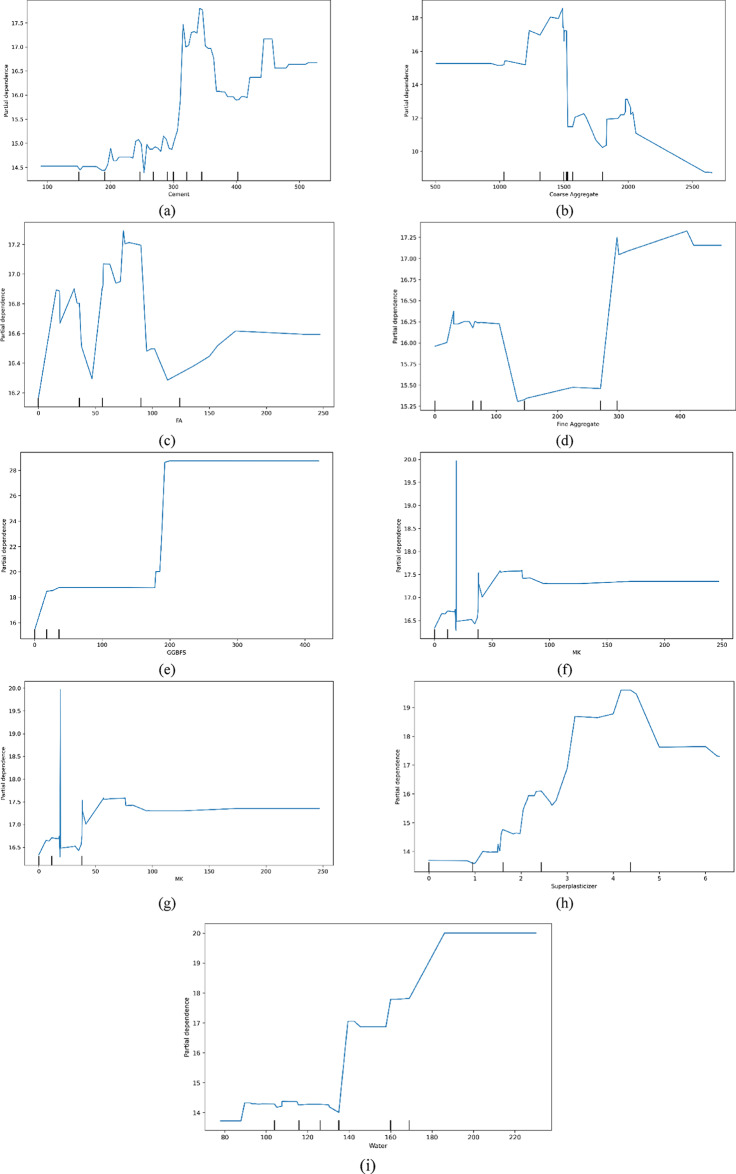
Examination of the stacking model through PDP for the input variables.

Figure 15 illustrates the impact of various input factors on the compressive strength of concrete. In Fig. 15(a), an increase in cement content from 300 to 375 kg/m^3^ correlates with a rise in compressive strength. Figure 15(b) shows that coarse aggregate contributes positively to compressive strength, peaking at 1500 kg/m^3^ before declining from 20 MPa to 10 MPa. The partial dependence plot (PDP) in Fig. 15(c) indicates that fly ash (FA) has a negligible effect on compressive strength. Conversely, Fig. 15(d) reveals that fine aggregate substantially increases compressive strength when its quantity exceeds 275 kg/m^3^, with values climbing from 15.5 MPa to 17.25 MPa. Figure 15(e) highlights the favorable impact of ground granulated blast furnace slag (GGBFS), where compressive strength rises from 18 MPa to 28 MPa as GGBFS content increases from 150 to 200 kg/m^3^, stabilizing beyond 200 kg/m^3^. The influence of metakaolin (MK) is depicted in Fig. 15(f), where its effect becomes significant at around 25 kg/m^3^. Figure 15(g) illustrates that silica fume (SF) enhances compressive strength up to 30 kg/m^3^, after which its impact stabilizes. Finally, Figs. 15(h) and (i) show that superplasticizer (SP) gradually improves compressive strength up to 5 kg/m^3^ before declining, while water plays a crucial role in compressive strength within the range of 140 to 190 kg/m^3^. The performance of RHA-FA concrete is considerably enhanced by the optimal range for enhancing compressive strength, which is achieved with a fly ash content of 20–30% and rice husk ash proportions below 10%, as revealed by PDP analysis^108^.

## Conclusions

This study aimed to assess the reliability of AI models in predicting the compressive strength of concrete and to examine the effects of various raw materials on compressive strength. Nine critical input factors were evaluated for their predictive power, and their interrelations were thoroughly investigated. The outcomes of this research led to the following conclusions:

- All five machine learning algorithms (XGB-LGB, XGB-DT, XGB, LGB, and DT) exhibited robust predictive capabilities and a strong ability to generalize. With R^2^ values surpassing 0.89, these models proved highly efficient in forecasting concrete strength, demonstrating their dependability for precise predictions.
- The ensemble ML models, XGB-LGB and XGB-DT, provided enhanced performance compared to the single models, showcasing improved accuracy and effectiveness. Furthermore, the hybrid models reduce overfitting risk by minimizing the difference between training and testing R^2^ value.
- Among all the single ML models, DT exhibited the top performance, with the lowest RMSE of 2.863, MAPE of 16.641, and MAE of 1.941. Furthermore, the hybrid XGB-LGB model demonstrates strong balanced performance with a slightly better correlation than other models.
- The SHAP analysis revealed that coarse aggregate, superplasticizers, water, GGBFS, and cement were the most significant elements impacting the predicted compressive strength. Additionally, the PDP evaluation indicated that the best mixture for optimizing concrete strength comprised 150–200 kg/m^3^ of GGBFS.

The established methods could assess the compressive strength of industrial waste-based concrete utilizing a variety of input variable values, saving time and effort for further experiments. Implementing these algorithms on a construction site speeds up work and reduces the need for time-consuming laboratory testing.

## Limitations and further studies

Although it is vital to note that other elements, such as the chemical and physical qualities of materials, fluctuations in temperature, and resistance to corrosion, chloride, and acid attacks, could potentially influence the compressive strength of industrial waste-based concrete, it is also important to make sure that these other factors are taken into consideration. That being said, this study was unable to conduct a complete analysis of the impact of these characteristics because there was a limited amount of data available. Consequently, the objective of future research should be to broaden the scope of the database so that it incorporates these additional parameters. This will make it possible to construct more robust predictive models that are based on deep learning. In addition, additional research could investigate the possibility of applying the machine learning frameworks that have been developed for the purpose of forecasting other essential qualities of concrete. These properties include flexural strength, tensile strength, durability, porosity, flowability, shrinkage, cost, and carbon footprint.

## Data Availability

Our objective is to maintain control over unsupervised usage that may lead to unintentional duplication of research efforts or reduced novelty in future studies. however, the dataset will be provided upon request. Please contact Dr. Md. Habibur Rahman Sobuz (email: habib@becm.kuet.ac.bd) if anyone needs the data for this study.
